# A simple compound prioritization method for drug discovery considering multi-target binding

**DOI:** 10.1039/d5dd00464k

**Published:** 2026-02-10

**Authors:** Alžbeta Kubincová, David L. Mobley

**Affiliations:** a Department of Pharmaceutical Sciences, University of California Irvine Irvine CA 92697 USA dmobley@uci.edu

## Abstract

Active learning is an emerging paradigm used to help accelerating drug discovery, but most prior applications seek solely to optimize potency, whereas multiple properties influence a compound's utility as a drug candidate. We introduce a method for multiobjective ligand optimization, which is able to efficiently handle distinct molecular properties that are expensive to compute, such as binding affinities with respect to multiple protein targets. We validate this protocol retrospectively using docking scores, showing an improved retrieval of the top 0.04–0.4% binders from the dataset with our method compared to greedy acquisition, owing to a better distribution of the compute budget between different properties. Our results also suggest that fitting individual properties separately leads to a better rank correlation of the resulting predictions. This workflow addresses the needs of pharmaceutical research for improving the efficiency of hit-to-lead and lead optimization by considering binding to multiple targets. Our code is freely available on Github: https://github.com/MobleyLab/active-learning-notebooks/blob/main/MultiobjectiveAL.ipynb.

## Abstract 2

Active learning is an emerging paradigm used to help accelerating drug discovery, but most prior applications seek solely to optimize potency, whereas multiple properties influence a compound's utility as a drug candidate.

## Graphical abstract

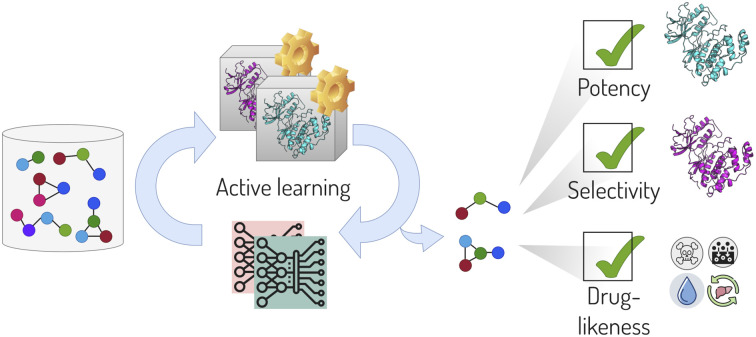

## 1 Introduction

**Fig. 1:**
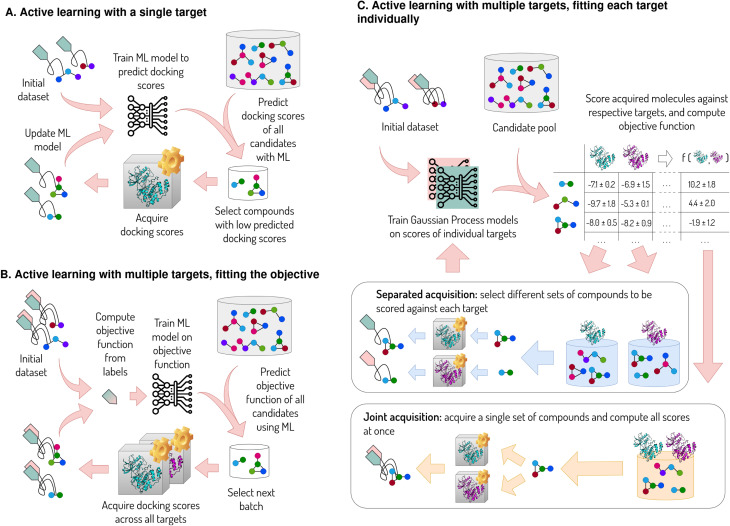
Single- and multiobjective optimization algorithms considered in this work. A small sample of a ligand set is scored, and a surrogate ML model (Gaussian process regression in this work) is trained on these compounds to predict the scores of a larger set, which is usually orders of magnitude faster than computing the scores. The predictions from ML are used to select a small batch of compounds for scoring, and the cycle is repeated multiple times. (A) Algorithm for optimizing the docking score (green tag) towards a single target. (B) Optimization of the ligand utility, which is encoded by an objective function composed of docking scores towards multiple receptors (green and pink tags for the two sets of scores). The workflow is technically identical to the single-objective case, with the utility function being used in place of a single property. (C) Optimization of ligand utility, while training a separate ML model for each target property. The acquisition can either be guided by the individual properties predicted for each molecule (separated acquisition) or by the overall objective function (and its uncertainty) computed from the predicted scores (joint acquisition).

Active learning (AL) strategies for identifying molecular drug candidates gained significant attention in the pharma industry over the last decade. Instead of scoring large chemical libraries with expensive physics-based methods, a surrogate machine learning (ML) model is trained on a small subset of scores from the expensive method, and used to predict the scores of a much larger compound dataset. These predictions are used to select next batch of compounds using an acquisition function, which links the predicted scores and their uncertainties to selection priority. The selected compounds are then scored with the physics-based model, and the ML model is updated with the new scores to improve its predictive performance (Fig. 1A). This approach allows for scanning large chemical spaces using only a fraction of the computational cost associated with scoring of the whole library in a brute force manner, while also building and improving a predictive model.^1–3^

The application of AL to computational drug design is still in its early stages, and most studies to date only optimize for potency, estimated from docking scores^4,5^ or relative/absolute binding free energies (RBFE/ABFE) from molecular dynamics (MD) simulations.^6–9^ However, the utility of a molecule as a drug candidate is influenced by properties other than the potency, particularly by ADME (absorption, distribution, metabolism, and excretion) properties, toxicity and selectivity. Some AL studies account for ADME properties by applying molecular property filters upon constructing the ligand dataset^6,9,10^ to remove compounds from the pool that are likely to have poor ADME properties. On the other hand, compounds that are potentially useful and fall slightly short of the ideal property ranges are discarded by this procedure, whereas a smooth penalty can account for trade-offs between properties.^11^

However, a filtering approach is rarely desirable to account for toxicity and selectivity. While the ranges for ADME properties are usually well-defined, drug safety is measured as the ratio of potency and toxicity (therapeutic index).^12^ In a typical drug discovery campaign, selectivity is only assessed for compounds with good potency,^13^ using separate free-energy calculations. The staged approach for on- and off-target free energy calculation reduces the number of expensive MD simulations, but can miss regions in chemical space, with slightly lower potency but better safety. Compounds found in lead optimization entering the preclinical phase are therefore often fraught with liabilities that are hard to mitigate. More than 90% of compounds entering clinical trials fail, with 10–15% due to poor drug-like properties and 30% due to toxicity.^14^ It might therefore be advantageous to explicitly account for off-target effects during optimization rather than as a filtering stage, especially in a streamlined early discovery process with AL.

To integrate multiple properties directly, researchers can either define a compound's utility using a single overall objective function combining these properties or use Pareto optimization. An objective function defines the relationship of each property to a drug's utility.^15,16^ The construction of an objective function requires the user to define a weighting scheme for the individual properties, which is not straightforward and involves trial and error.^17^ On the other hand, Pareto optimization considers properties as separate objectives to optimize. A compound is Pareto optimal if none of its properties can be improved without degrading another property. This method does not require a weighting scheme and is often preferred for its stability and consistency.^18–21^ On the other hand, Pareto optimal solutions may not always reflect the needs of drug discovery (*e.g.* minimizing off-target binding at the cost of potency), and domain-specific knowledge may need to be included in a *post hoc* manner.^22^ In general, it is however not possible to directly compare the present scalarization of objectives with Pareto optimization, since their different definitions of the optimization target imply different optimization approaches and goals. Both methods at best only approximate the true utility of a compound as a drug molecule, as utility often depends on a particular balance of properties (which may not be known *a priori*) and may be influenced by other factors, such as structural novelty.

These approaches for multiobjective ligand optimization are well-suited to problems where at most one property is expensive to compute. Properties such as ADME can efficiently be precomputed and combined with free-energy estimates once they are calculated for a given compound. This is not the case for binding to multiple targets, where the affinity to each target requires a separate calculation that is expensive, especially if binding free energy calculations are used. All affinities need to be calculated at once to evaluate the objective function, which is used as a reward for the surrogate ML model (Fig. 1B). However, a subset of these affinities is often sufficient to discard a compound. Poor on-target potency eliminates the need for off-target screening. Likewise, if a dual inhibitor is sought, a weak binding affinity to one of the targets means there is no need to calculate potency towards the second target since the compound can already be ruled out. Therefore, a simultaneous calculation of multiple properties only makes sense if the prior distributions of all properties (*e.g.* predictions from an ML model) are in a favorable range and sufficiently narrow (*e.g.* good potency and selectivity are predicted with high confidence). However, in case of high uncertainty, the compute budget would be better split across potency calculations for multiple compounds rather than exhausted on computing all off-target affinities for a single compound that may not be potent. This can become an issue for targets with many similar analogues (for example, the selectivity of a JNK1 inhibitor series was affirmed upon screening against a panel of 74 kinases^23^).

In this work, we introduce an AL method that efficiently accounts for multiple properties that are expensive to compute (Fig. 1C). The method relies on an objective function for a given problem, but acquires different batches of compounds for the computation of each property (separated acquisition) using a modified version of the Expected Improvement (EI) acquisition function^24,25^ (see the Theory section). The separated acquisition allows for a better distribution of the compute budget by taking into account each property's impact on the target candidate profile. The acquisition is separate from the ML model, and therefore does not stipulate any particular ML architecture as long as prediction uncertainties are available. The ML models are fitted to structural features of molecules to predict their properties. Here, we use a separate Gaussian process model to fit docking scores with respect to each target. The protocol can also be described as an independent AL cycle for each property, with the planner communicating upon acquisition of new compounds.

The goal of this work is to create a framework for the optimization of potency in the presence of off-targets or for multi-target inhibition. We develop and validate this framework using docking scores as a proxy for binding affinity, although more accurate and expensive methods will be required in real applications. The use of docking scores here, as we develop and test this framework, is motivated by docking scores' low cost. In particular, for our study here, they are available in large quantities for a suitable compound library while being derived from a consistent docking protocol. However, our framework also works in the same way using more expensive (and accurate) means of binding affinity prediction, such as free-energy calculations with MD, where these calculations dominate the computational (and monetary) cost of the workflow. Here, we do not devote significant attention to evaluating computational cost in terms of wall time. Specifically, since the framework is evaluated in a retrospective way using precomputed docking scores, wall times are not indicative of the performance in prospective applications in this case. We expect the potency calculation to be the slowest step in a prospective scenario, which is why we always compare between protocols that have access to the same number of potency samples for selecting the next batch of ligands.

Here, we demonstrate the superior hit rate of our separated acquisition strategy compared to joint acquisition on two optimization problems and provide insight into various parameters impacting hit retrieval.

## 2 Theory

In this section, we lay the theoretical framework for our acquisition strategy, which scores individual target properties rather than compounds as a whole. We provide a brief overview of the Expected Improvement acquisition function and adapt the function to separate the contributions of target properties.

### 2.1 Expected Improvement (EI) acquisition function

EI is a widely used strategy in Bayesian optimization to locate the maximum of an objective function *f*.^24,25^ Assuming that the probability density of *f* follows a normal distribution prior to measurement, 
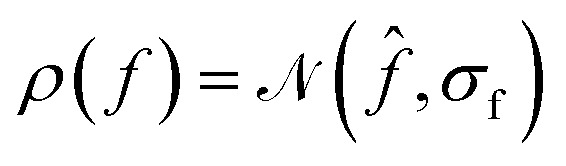
, EI is defined as the expected increase in the value of *f* over a reference value f* (usually set to the current maximum) following measurement (where we assume that the measurement is exact):1
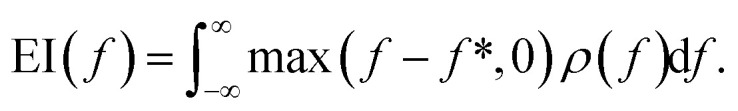
Eqn (1) evaluates to2
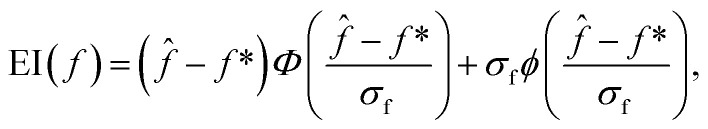
where *f̂* and *σ*_f_ are the mean and standard deviation of *f*, and *ϕ*(*x*) and *Φ*(*x*) denote the assumed probability density function and cumulative distribution function of *x*, respectively (here, for a normal distribution). In a drug discovery context, *f* stands for the objective function of a given compound, and eqn (2) would be evaluated for every compound in the pool.

### 2.2 Adapted EI for scoring individual properties

The original definition of EI, as given by eqn (1), measures the overall improvement of *f* for a given sample. We will now adapt EI to account for the improvement in *f* resulting from a given property *p*, which contributes to *f*. In practice, properties are chosen to reflect the target candidate profile of a drug, such as potency, selectivity and ADME, and the objective function defines their relationship to each other. This strategy is therefore different from Pareto optimization and the related Expected Hypervolume Improvement (EHVI) acquisition function.^26^ EHVI does not rely on an objective function, but instead measures the possible improvement of the Pareto front as a hypervolume in multivariate space. By contrast, our method relies on an objective function, but separates the contributions of the individual properties.

Consider a piecewise linear objective function *f* of multiple properties *p* ∈ *P*, defined in segments *s* ∈ *S* spanning the entire range of values that *f* can adopt. Consequently, in each segment, *f* can be written as a linear function of any individual property *p*, if all other properties *Q* = {*q* ∈ *P*|*q* ≠ *p*} are kept constant:

3 *f*(*p*|*Q*) = *α*_ps_(*Q*)·*p* + *β*_ps_(*Q*), *p*∈[*a*_ps_(*Q*),*b*_ps_(*Q*)].

*α* and *β* are the linear coefficients, and *a* and *b* are the bounds of each segment. To give an example, the objective function *f*(*A*, *B*) = min(*A*, *B*) can be written in this notation for the property *A* as *f*(*A*|*B*) = *A* if *A* < *B*, *B* if *A* > *B*. This results in two segments (*S* = 2), with the coefficients *α*_*A*1_ = 1, *β*_*A*1_ = 0 ranging from *a*_*A*1_ = −∞ to *b*_*A*1_ = *B* for the first segment, and *α*_*A*2_ = 0, *β*_*A*1_ = *B* ranging from *a*_*A*1_ = *B* to *b*_*A*1_ = ∞ for the second segment. Since the coefficients *α*, *β*, *a* and *b* can be functions of properties other than *p*, the terms from individual properties do not need to be additive. This allows one to consider scenarios where properties are coupled to each other, such as for the PPAR test system considered in this study (eqn (9)).

Next, we define the EI of a property *p* as the expected improvement of *f* over f* while optimizing only that single property *p*, while assuming the maximum likelihood estimate (MLE) for all other properties, *Q̂*:4
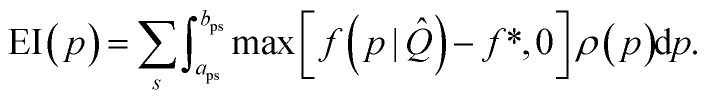
Eqn (4) is approximate owing to the MLE assumption, which avoids integrating over the joint probability distribution *ρ*(*P*) of all properties. This means that the sum of EI(*p*) terms over all properties will not add to EI(*f*). The MLE assumption has critical consequences in practice, which we will address later. However, the assumption is also crucial for practicality, as it breaks an integral over a multidimensional probability density down to a set of one-dimensional integrals, which can be computed analytically (in contrast to the EHVI, which is typically evaluated by Monte Carlo integration).

Substituting the definition of *f* from eqn (3) into (4), together with 
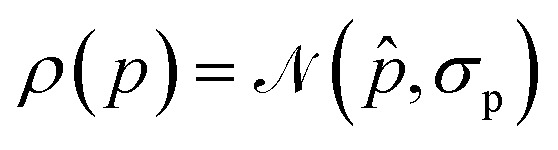
, results in5
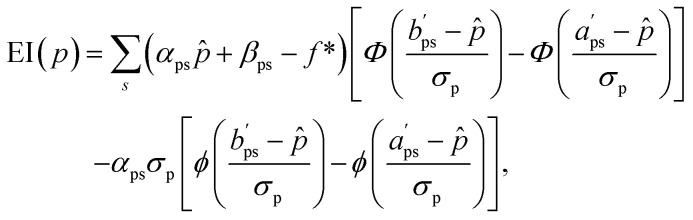
where 
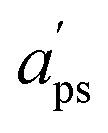
 and 
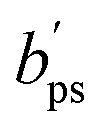
 are adapted from the bounds *a*_ps_ and *b*_ps_, ensuring that *f*(*p*|*Q̂*) − f* remains positive in the interval 
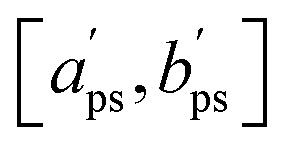
:6
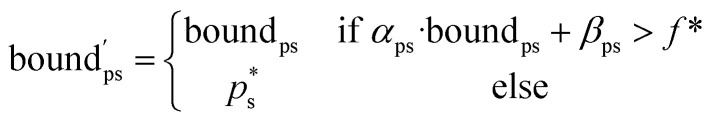
for bound ∈ {*a*_ps_, *b*_ps_} and 
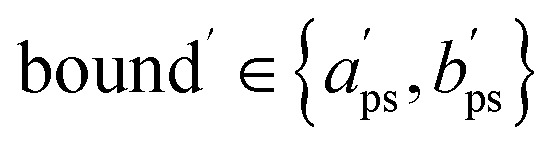
. 
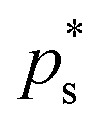
 is the value of *p* for which the segment function evaluates to f*, noting that 
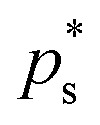
 may fall outside the interval [*a*_ps_, *b*_ps_],7
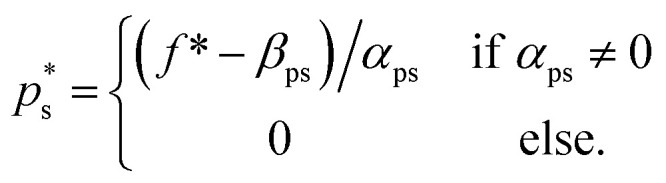
In the context of AL, out of the full library, a separate pool or subset of compounds is maintained for each property (*i.e.* docking score), and eqn (5) needs to be evaluated separately for every compound and property available for acquisition.

The Python implementation of EI(*p*) from eqn (5)–(7) is provided in the notebook included in the Github repository (see the Data availability). The user needs to provide the definition of *f*, as well as the coefficients *a*_ps_, *b*_ps_, *α*_ps_ and *β*_ps_ for each property and segment. The original EI formulation from eqn (1) can be recovered as a special case of eqn (5) with the parameters *a* = −∞, *b* = ∞, *α* = 1 and *β* = 0.

## 3 Methods

### 3.1 Benchmark design

We validate our active learning method using the DOCKSTRING dataset,^27^ considering discovery use cases where binding to more than one target influences a compound's utility. This dataset contains docking scores from 260 000 molecules against 58 clinically relevant targets and was constructed for the purpose of validating ML models for potency prediction. Ligand molecules were taken from the ExCAPE database,^28^ which curates bioactivity assays from PubChem and ChEMBL. The selection ensures that at least 1000 active molecules were selected for each target, together with 150 000 ligands with inactive labels against all targets. Experimental actives are expected to have better docking scores than inactives, although a good correlation between docking scores and experimental affinities cannot be guaranteed.^27^ The dataset was filtered to remove duplicate molecules (7 in total).

We encode the desirability of a ligand by an objective function to be maximized, which aggregates docking scores with respect to multiple targets. In addition to the docking scores, a penalty is added for molecules that are not drug-like, relying on the quantitative estimate of drug-likeness (QED)^29^ metric, which we compute using the 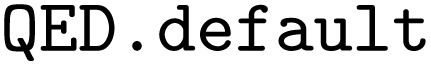 function from RDKit. The QED accounts for eight molecular properties (molecular weight, octanol–water partition coefficient, number of hydrogen bond donors, number of hydrogen bond acceptors, molecular polar surface area, number of rotatable bonds, number of aromatic rings and number of structural alerts), converting their desirability to a continuous scale ranging between 0 and 1. We consider two design objectives as suggested by the DOCKSTRING authors:^27^

(1) **Selective for JAK2**: this task involves maximizing the binding to JAK2, while minimizing binding to its off-target LCK. In order to avoid offsetting poor JAK2 binding by unusually weak LCK binding, the contribution from LCK is capped at its median score (−8.1).

8 *f*_JAK2_ = −JAK2 + min(LCK,−8.1) − 10(1 − QED).

**Fig. 2:**
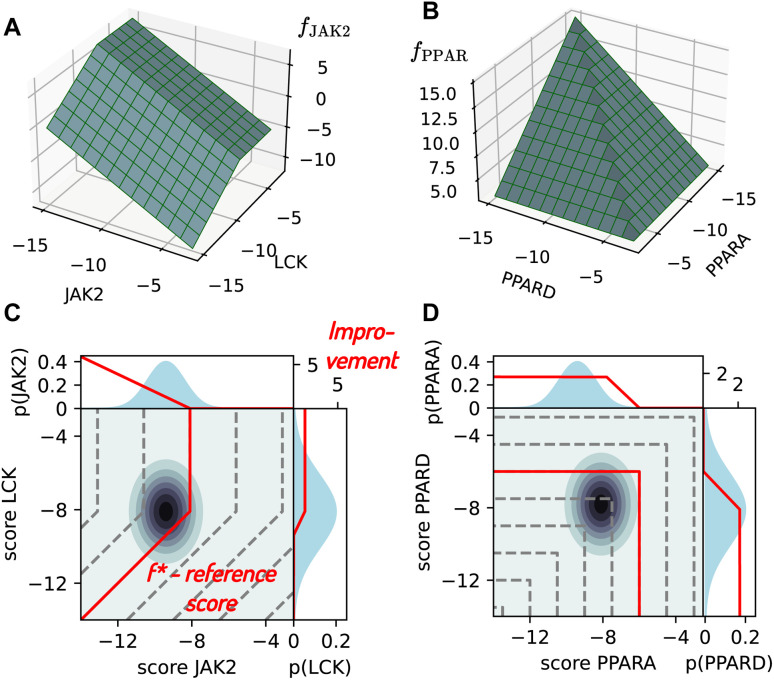
Visualization of objective functions for JAK2 and LCK, which aggregate docking scores across multiple targets. (A) *f*_JAK2_ from eqn (8) is shown as a function of JAK2 and LCK scores for QED = 1. (B) *f*_PPAR_ from eqn (9) is visualized as a function of PPARA and PPARD scores (assuming PPARG = −∞ and QED = 1). (C) Contour plot of *f*_JAK2_ with a representative data point predicted with a mean and uncertainty for both JAK2 and LCK. Marginal probability distributions are shown for both properties (blue curves) together with their improvement max(*f*(*p*|*Q̂*) − f*,0) in red. The blue and red lines represent the components of the property-specific EI integral from eqn (4). (D) Contour plot for *f*_PPAR_, in analogy to (C).

Fig. 2A shows a graphical representation of *f*_JAK2_ as a function of JAK2 and LCK. Eqn (8) offers the advantage of simplicity, but other functional forms can be used to account for selectivity in practice. In particular, a penalty for the difference between on- and off-target binding may better represent the need for a therapeutic window, rather than anchoring off-target scores to a fixed value.

(2) **Promiscuous for PPAR**: this task involves finding inhibitors simultaneously binding to three PPAR receptors, namely PPARA, PPARD and PPARG. The objective function therefore maximizes the potency towards the weakest-binding receptor and is visualized in Fig. 2B:

9 *f*_PPAR_ = −max(PPARA,PPARD,PPARG) − 10(1 − QED).

### 3.2 Multiobjective AL workflow

The workflow (Fig. 1B and C) is seeded with an initial set of 1000 randomly selected ligands, for which all properties are acquired at once. The size of the initial dataset was chosen upon confirming that all Gaussian process models fitted to these data are strongly predictive (Spearman *ρ* = 0.6 at minimum). We chose the initial batch size in accordance with comparable studies by the DOCKSTRING authors,^27^ and we did not optimize batch sizes in our study. In practice, the amount of samples for training a predictive ML model varies greatly with the underlying data^30^ and is particularly dependent on the structural diversity within the dataset and the strength of the dataset's structure–activity relationships. In case of the JAK2 test system, we observed similar outcomes when seeding the workflow with only 100 ligands (SI Section 2).

The ligands are featurized using chiral Morgan fingerprints as implemented in RDkit with a radius of 4 and a length of 1024 bits. We chose this feature set based on its widespread use in the literature and because we are aware of molecules in DOCKSTRING that differ only in their stereochemistry. However, we did not optimize our feature selection with respect to the predictive accuracy on this dataset, in order to reflect an application scenario where insufficient data are available to carefully select a high performing feature set. A comparison of Morgan fingerprints with MACCS keys is provided in Section 1 of the SI, showing that concatenating these two representations can further improve the predictive accuracy of the ML model.

One or multiple Gaussian process (GP) models were fitted to the reward given by either the objective function or individual docking scores. The GP was chosen for its ability to natively provide prediction uncertainties and for its good performance with small amounts of training data. The model was implemented using GPyTorch with a Tanimoto similarity kernel. In each cycle, the budget for acquisition was fixed at 300 scores for the separated acquisition strategy, which translates to 150 and 100 compounds with the joint acquisition strategy for JAK2 and PPAR, respectively. In total, we acquire about 1% of all DOCKSTRING ligands for JAK2 and 0.8% for PPAR, taken together from the initial batch and the subsequent 10 optimization cycles. Five repeats are executed for each scenario with different random seeds for the initial batch selection, in order to calculate the uncertainty of overall metrics. We also show that it is possible to use this protocol with smaller batch sizes in Section 3 of the SI.

### 3.3 Training and acquisition strategies

The separated acquisition strategy given by eqn (5)–(7) requires individual docking scores to be available for pool compounds, rather than only the objective function *f* (since docking scores are treated as separate properties *p* in eqn (5)). This strategy therefore necessitates training individual ML models for each target property. In order to evaluate training one or multiple ML models separately from the acquisition strategy, we consider three major versions of the optimization procedure: joint training and acquisition, separated training with joint acquisition, and fully separated training and acquisition.

The joint training and acquisition strategy relies on a single ML model, which is fitted to *f* directly, as shown in Fig. 1B. The workflow is technically identical single-objective optimization (Fig. 1A), where the objective function is used as an optimization objective in place of a single affinity score. Docking scores acquired for the initial dataset as well as subsequent compound batches are converted to the objective *f*, which is used as a reward for training the ML model. Consequently, inference on the compound pool and acquisition of the new batch is only informed by *f* rather than individual properties. We implement two acquisition strategies: EI based on eqn (2), and a greedy strategy selecting compounds with the highest predicted score *f*.

Another strategy involves the training of separate ML models for each property while acquiring new compounds based on *f* computed from individual properties (Fig. 1C). In this case, individual ML models are fitted to each docking score separately, rather than to *f*, and the QED is precomputed for the whole dataset (which only takes a few minutes). Upon inference, *f* is computed from the predicted properties, with their uncertainties propagated onto *f* according to10
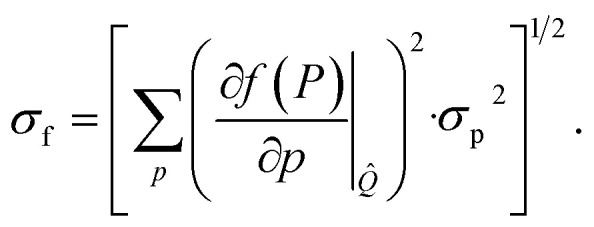
Here, we also apply EI from eqn (2) as well as a greedy acquisition strategy.

Lastly, we consider a setup with separate ML models coupled with the separated acquisition strategy (Fig. 1C). In this case, the acquisition is informed by the individual docking scores rather than *f*, and different batches of compounds can be acquired for each target property. The acquisition is implemented as follows; For each compound–property pair that has not been measured yet, EI(*p*) is calculated using eqn (5)–(7). The values are sorted, and compound–property pairs with the highest EI(*p*) are acquired, where the batch size is defined as the number of scores, or compound–property pairs. This strategy adds flexibility to efficiently distribute the acquisition budget across different targets as a response to the objective function as well as the underlying data (*cf.*Fig. 2C and D). However, the acquisition strategy does not guarantee that valuable compounds will be scored against all targets, which is why we switch to a greedy strategy for the last cycle, acquiring (missing) scores of compounds with the highest predicted value of *f*.

In summary, five different strategies are evaluated:

• **EI joint**: joint training with joint acquisition based on EI.

• **greedy joint**: joint training with joint acquisition based on a greedy strategy.

• **EI sep. train.**: separated training with joint acquisition based on EI.

• **greedy sep. train.**: separated training with joint acquisition based on a greedy strategy, and

• **EI sep. acq.**: separated training with separated acquisition using the modified EI algorithm.

All of the strategies outlined above are exploitative, aiming to acquire potent ligands rather than enhancing the accuracy of the underlying ML model(s). Instead of adding rounds of explorative acquisition, we rely on the models built on the initial batch to be predictive enough to assist in exploitation. In some cases, balancing exploration with exploitation can be important in active learning, especially with regard to retrieving diverse hits and improving quality of the model. However, these considerations – and the possibility of balancing exploration *vs.* exploitation – are beyond the scope of the present work, where the number of hits is used as the primary metric for comparing between optimization protocols.

There is one key difference in our calculation of the EI compared to its original definition of f*. The value of f* can be interpreted as an acquisition boundary, such that the improvement from possible values of *f* below f* is zero. Although this parameter is traditionally defined as the best (true) score among the acquired compounds, it is treated as a constant in eqn (1)–(7) and can therefore be set to any other (constant) value without loss of generality. Here, to avoid problems where acquisition might get blocked when further score improvement requires optimization of multiple properties, we apply a different strategy. Specifically, we set f* such that f* < *f̂* for any compound in the pool, which would be acquired by a greedy strategy in the current cycle. For the joint acquisition strategy, f* is set equal to the predicted score of the top 150th and 100th compound in the pool for JAK2 and PPAR (since 150 or 100 compounds are acquired in each cycle), whereas separated acquisition relies on the top 300th score (since the scores can be distributed freely across different properties, which may result in acquiring a single property for 300 compounds). We introduce this modification because separated acquisition does not perform well with the original choice of the f* boundary. The improvement EI(*p*) can only be greater than zero if the value of *f* can increase by changing any single property at a time (*i.e.* an upward move is possible along the dark green lines in Fig. 2A and B). This is not always the case for properties, which only contribute to *f* up to a limit (LCK in eqn (8), and all of the PPAR receptors in eqn (9)). Therefore, it is possible for a compound to have a high EI(*f*) (resulting in acquisition by the joint strategy), but EI(*p*) = 0 for all properties (resulting in non-acquisition by the separated strategy). This behavior is a consequence of the MLE approximation in eqn (4). The alternative choice of anchoring f* to the predicted scores of pool compounds circumvents this issue, ensuring that all compounds that would be selected by a greedy strategy fulfill EI(*p*) > 0 for all properties. We will show in Section 4.4 how the choice of f* affects the retrieval of desirable compounds.

## 4 Results and discussion

### 4.1 Separating training and acquisition boosts the retrieval of top binders

**Fig. 3:**
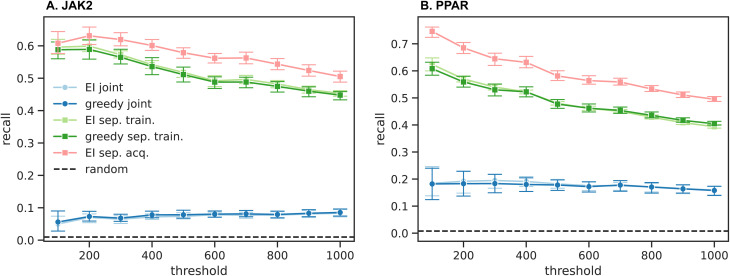
Fraction of active compounds retrieved for the JAK2 (A) and PPAR (B) systems, as a function of the threshold rank for classifying a compound as active. The recall is calculated as the number of actives found within the 10 cycles, divided by the corresponding threshold. Lines represent the different strategies for training and acquisition as outlined in Section 3.3. In case of separated acquisition, the recall is based on compounds that had all of their properties acquired. These data show that training separate ML models has a distinct advantage over fitting the objective function directly (specifically, all of the curves with separate training are far higher than those with joint training), whereas no significant difference can be seen between EI and greedy acquisition. The separated acquisition strategy offers an additional advantage for both systems, especially for PPAR.

The five optimization protocols are compared based on the number of hits retrieved within 10 cycles, measured by the objective functions in eqn (8) for JAK2 and eqn (9) for PPAR. Fig. 3 shows consistent trends between different protocols for JAK2 and PPAR. Here, we define an active compound as one with a rank higher than a given threshold, considering thresholds ranging from 100 to 1000. The recall is then computed for a given threshold as the fraction of active compounds retrieved within 10 cycles (*i.e.* retrieving 60 out of the top 100 compounds according to *f* results in a recall of 0.6 for the threshold 100). The thresholds considered account for the top 0.04–0.4% of the whole dataset, and all protocols show a clear advantage in recall over a random selection in this range of thresholds (random selection results in a recall of <0.01 for both systems).

Comparing individual protocols reveals that the training as well as acquisition strategies significantly impact the number of hits retrieved, whereas no significant difference can be seen between greedy and EI acquisition. The largest improvement in hit retrieval is achieved by fitting separate models for each property, rather than fitting *f* directly. Separated acquisition does however offer an additional advantage in almost all cases. We will rationalize these findings in more detail in the following sections.

The docking scores of JAK2 and LCK are positively correlated, as are the scores of the three PPAR receptors.^27^ While the correlation of scores increases the difficulty of selectivity optimization, it simplifies the task of finding promiscuous inhibitors in most cases. A unified ML model can better account for features that enhance the binding to all PPAR receptors at the same time, in contrast to learning miniature differences between on- and off-target binding. The separated training strategies show comparable trends between JAK2 and PPAR, since each model learns distinct structure–activity relationships for each target. It is however not clear how the correlation between scores affects the separated acquisition trends, as the improvement in PPAR recall may also be influenced by the higher flexibility for distributing the acquisition budget across three, rather than only two, receptors.

### 4.2 Training separate models for individual properties improves model predictivity

**Fig. 4:**
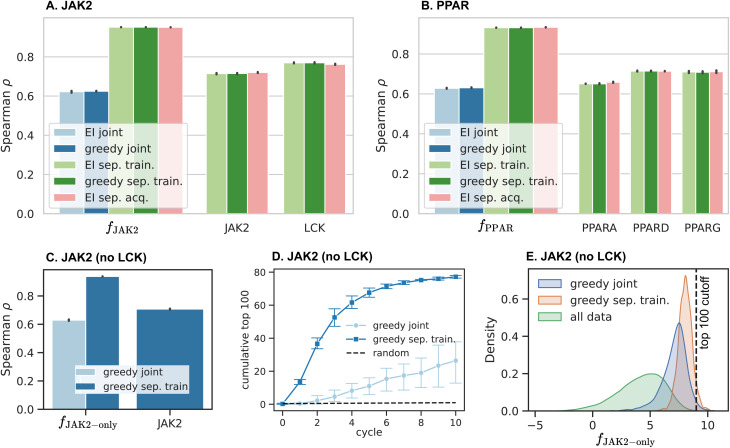
Spearman's rank correlation of the docking scores predicted in the last cycle with their true values and their effect on acquisition. (A) Rank correlation of the predicted objective function *f*_JAK2_ for the five protocols, as well as the correlation of scores predicted by the target-specific models for JAK2 and LCK. (B) Rank correlation for the models fitted to PPAR scores, in analogy to (A). (C) Correlation of the objective function *f*_JAK2-only_ and JAK2 predictions in a JAK2-only optimization, eqn (11). (D) Retrieval of the top 100 compounds measured by *f*_JAK2-only_. (E) Probability density of the objective *f*_JAK2-only_, calculated for the compounds retrieved within 10 cycles (excluding the initial batch), compared to the *f*_JAK2-only_ distribution of all ligands in DOCKSTRING. These data show that models trained on *f* directly have an inferior rank correlation compared to models fitted separately to each target, which is mainly caused by the QED being fitted together with docking scores for the joint models.

To understand the relationship between recall and model predictivity, we compare the models from each protocol based on their Spearman rank correlation *ρ* between properties predicted in the final cycle with the true values for these same properties (Fig. 4A and B). While all models are strongly predictive, computing the objective function from individual docking scores predicted by separate models improves *ρ* by a factor of 1.5 compared to predicting *f* directly. On the other hand, the acquisition strategy does not influence the model predictivity in any significant manner.

Two factors contribute to the performance gap of separated and joint ML models. First, being trained on a combination of docking scores towards multiple targets, the joint models need to learn multiple structure–activity relationships at the same time as well as their relationship to each other, making this a difficult problem for training. Second, the joint models are required to learn drug-likeness (QED) simultaneously with the docking scores, whereas the QED is treated as a lookup with the separated training strategy, since the QED computation is fast and does not warrant being treated as a separate property for training and acquisition in practice. To assess the impact of including the QED into the training objective in the absence of other targets, we consider a simplified version of the JAK2 problem by eliminating LCK as an off-target:

11 *f*_JAK2-only_ = −JAK2 − 10(1 − QED).

The correlation of final models from optimizing *f*_JAK2-only_ from eqn (11) in Fig. 4C shows a similar trend to JAK2 and PPAR regarding the separated models outperforming the joint ones by a good margin. This suggests that the QED is the primary factor impacting model predictivity. On the other hand, any of the models trained on a distinct target outperforms the corresponding joint model, suggesting that individual docking scores may be easier to fit compared to the aggregated objective. The importance of improving the underlying model for finding hits can also be understood from Fig. 4D and E. Although the joint training strategy achieves two thirds of the ranking performance compared to separated training, it only retrieves one third of the top 100 compounds in comparison (Fig. 4D), since the recall is determined by the tail of the score distribution of acquired compounds, which is very sensitive to small improvements of the model (Fig. 4E).

Despite the improved fit, we must also note that training and predicting from separate ML models increases the computational cost. The overall computational cost will only be comparable if the calculation of binding scores is orders of magnitude more expensive than the cost of training and inference. Maintaining separate models may not be desirable if the costs of training and obtaining labels are comparable, and multi-task learning approaches may be preferred.^31^ We did not pursue this approach here for the sake of simplicity.

### 4.3 Separated acquisition adapts to the objective function as well as to the underlying data

In the last Section, we have shown how the model predictivity impacts the hit rate in order to rationalize the superior recall of the separated training protocols. In this Section, we relate the improvements in hit rate from the separated acquisition to the distribution of the acquisition budget across different properties.

**Fig. 5:**
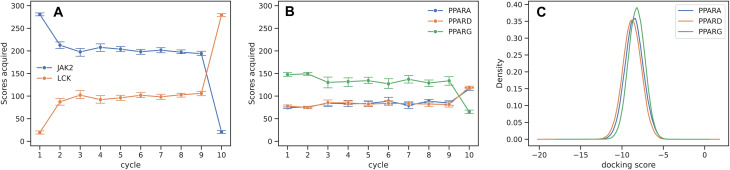
Number of scores acquired of each kind using the separated acquisition strategy for JAK2 (A) and PPAR (B). Protocols relying on joint acquisition translate to 150 scores per cycle of JAK2 and LCK each, and 100 scores for each of the PPAR receptors. The acquisition strategy changes in the last cycle to a greedy one for acquiring missing properties of high-scoring compounds. (C) Probability density of docking scores for the three PPAR receptors in the dataset. The preference for JAK2 acquisition over LCK can be explained by different contributions of their scores to *f* (Fig. 2C). For PPAR, PPARG is acquired more than the other two receptors due to weaker binding of the ligands on average (C).

Fig. 5 shows the number of docking scores for each target acquired per cycle by the separated acquisition strategy for JAK2 (Fig. 5A) and PPAR (Fig. 5B), given a budget of 300 scores per cycle. Both test systems result in an unequal allocation of the score budget compared to the joint acquisition strategies, which are bound to acquire 150 scores of each kind for JAK2, and 100 for each of the three PPAR receptors. The dominant acquisition of JAK2 scores over LCK can be understood from the contributions of both properties to the objective function from eqn (8). *f*_JAK2_ scales linearly with JAK2 potency, but is insensitive to LCK beyond a maximum value, which is why EI(JAK2) is usually larger than EI(LCK) (Fig. 2C). The acquisition function responds to the design of the objective function by favoring the more impactful variable, which is JAK2. In case of PPAR, on the other hand, the objective function from eqn (9) is symmetric with respect to the three receptors, suggesting no preference for any one of them. The emphasis on PPARG acquisition seen in Fig. 5B is probably related to the distribution of the docking scores for the three receptors, which is shown in Fig. 5C. PPARG docking scores are higher (*i.e.* worse) on average compared to PPARA and PPARD, thus having the greatest potential for improving *f*_PPAR_. This analysis showcases the adaptability of the separated acquisition strategy towards the objective function, as well as to the underlying data. The separated acquisition protocol eliminates the need for user-defined protocols based on manual inspection of the target candidate profile and existing data, especially since this task increases in difficulty for more complex objectives and a growing number of on- and off-targets.

### 4.4 Anchoring the acquisition boundary to the pool is crucial for separated acquisition

As described in Section 3.3, our definition of EI differs from the original formulation in the choice of the reference value f*. The original definition of EI relies on the current maximum of *f* from among the acquired data, whereas we anchor f* to the compounds, which are still in the pool, such that any compound that would be acquired by a greedy strategy fulfills *f̂* > f*. In the following, we compare protocols for different definitions of f* in order to illustrate why we made this choice.

**Fig. 6:**
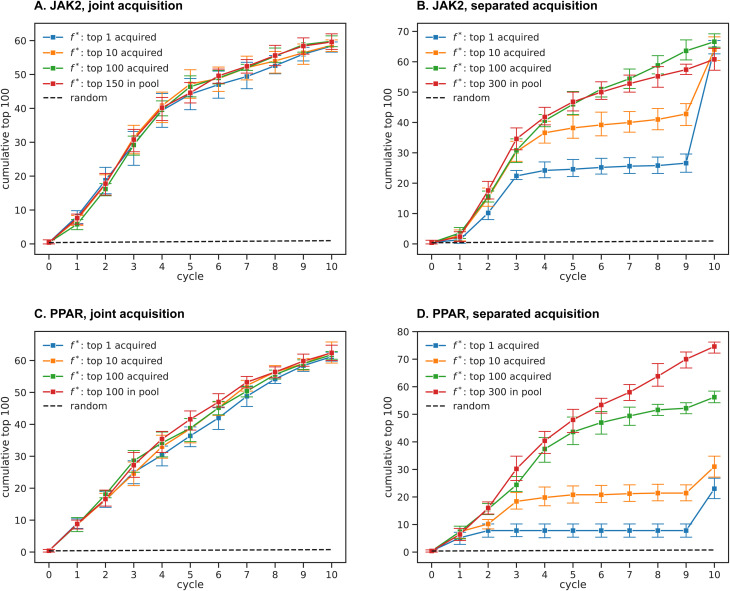
Comparison of acquisition protocols derived from EI with different choices for f*, coupled with separated models for each property. The figures show the recall of top 100 compounds according to the corresponding objective function for JAK2 and PPAR. f* is the reference value for computing improvement, which can be either set relative to *f* of the acquired compounds, or relative to the predicted value of *f* for compounds, which are still in the pool. The original EI definition is reflected by the blue lines, and our modified EI, which was used in the rest of the study, is shown in red. (A) Joint acquisition based on eqn (2) for JAK2. (B) Separated acquisition based on eqn (5) for JAK2. (C and D) Analogous to (A) and (B) for PPAR. It can be seen that anchoring f* to the pool compounds is beneficial for separated acquisition, whereas the choice of f* does not significantly impact joint acquisition.

Fig. 6 shows the number of top 100 compounds retrieved within a given number of cycles for different choices of f*, using both the joint (Fig. 6A and C) and separated (Fig. 6B and D) acquisition strategies. It is immediately visible that the recall is not significantly altered by changing f* for joint acquisition, while having a strong impact on separated acquisition. If f* is anchored to the acquired compounds, the recall flattens out after a few cycles, once most of the compounds with *f* > f* have been acquired. In case of JAK2 (Fig. 6B), shifting f* to higher values (and lower ranks) leads to acquiring more JAK2 scores and fewer LCK scores, resulting in a lower recall. However, all protocols converge in the last (greedy) cycle, which acquires the missing LCK scores. This is not the case for PPAR, where the protocols diverge, and setting f* relative to the acquired compounds drastically impedes retrieval of high-scoring hits (Fig. 6D). The problem with PPAR can be understood from its objective function in eqn (9), where each of the scores can improve f* only up to a limit imposed by the two other scores. In this case, improvement would only be possible from acquiring two or more of the scores at the same time, and this kind of improvement cannot be computed within this framework, which only considers properties in isolation. In the pathological scenario where PPARA = PPARD = PPARG = f* − *δ*, EI = 0 for all three receptors even though the probability of improving f* is roughly 1/8 (if all uncertainties are equal). Therefore, this compound will never be acquired, even if it was the second highest scoring compound in the dataset. Setting f* relative to the pool eliminates this problem (see Section 3.3), since f* now changes at every cycle according to the predictions for the top candidates for acquisition. This choice of f* results in a higher number of hits retrieved for PPAR, compared to both the original definition of EI, as well as the joint acquisition. We have not tested ranks different from 300 for selecting f* relative to the pool, and the protocol may further be improved by adjusting this parameter.

## 5 Conclusion and outlook

We introduced an AL strategy for multiobjective ligand optimization using an acquisition function, which can efficiently handle multiple properties that are expensive to compute. We retrospectively validated our method on two toy problems from the DOCKSTRING benchmark, showing that our separated acquisition protocol retrieves more hits compared to joint acquisition. The separated acquisition strategy, which is based on EI, takes advantage of imbalances in the contributions of each property to the objective function, as well as imbalanced distributions of the individual properties in the underlying data in order to rule out compounds without acquiring all of their properties. Our results also suggest that individual docking scores are better fitted with separate ML models rather than training on the objective function directly. Building separate models results in predictions that better correlate with ground truth values, as well as an improved recall of top compounds.

The method is straightforward to implement and scalable with the number of properties (receptors) and is therefore expected to perform well for more complex utility functions involving multiple on- and off-targets or properties that may not directly relate to target binding. Since this framework can take advantage of any kind of ML model, it can potentially incorporate other architectures, keeping pace with rapid advancements in this field. In particular, multi-task learning is a promising avenue for a growing number of targets to keep the costs associated with training and inference low.

Limitations of our approach include the requirement of an objective function as an input, the lack of consideration for exploration and compound diversity, and the need for prediction uncertainties. The need for an objective function is inherent to the method and cannot easily be eliminated, and the outcome might be susceptible to the weighting of the individual properties. On the other hand, exploratory aspects can easily be incorporated into the workflow, *e.g.* by means of clustering compounds to promote the selection of different scaffolds, which was shown to be effective in previous work.^7^ Additionally, although our framework is agnostic with respect to the ML architecture, most ML models do not natively predict uncertainties, which the framework relies on. This need can however be addressed with a range of model-agnostic uncertainty quantification metrics.^32^ These limitations will be addressed in future work.

To showcase the utility of our method in computer-aided drug design, we intend to apply it prospectively to a discovery problem, which requires balancing the binding to multiple targets, by using free energies from MD as a measure of potency. We hope that the consideration of potential liabilities early in the discovery process will increase the likelihood of the found hits to become successful drug candidates.

Our protocol can easily be combined with generative design strategies. This can be achieved by filtering the designs to a small sample enriched in binders to multiple targets, and in turn using the selected compounds to fine tune the generative model. This approach can be executed in successive stages, allowing for a better exploration of chemical space.^33^ The use of a scalar objective function makes our protocol compatible with a wide range of *de novo* design packages, even those that do not support multi-objective rewards (such as REINVENT^34^).

## Author contributions

A. K.: conceptualization, methodology, software, validation, formal analysis, investigation, data curation, writing – original draft, writing – review & editing, visualization. D. L. M.: resources, writing – review & editing, supervision, project administration, funding acquisition.

## Conflicts of interest

D. L. M serves on the scientific advisory board of OpenEye Scientific Software, Cadence Molecular Sciences and is an Open Science Fellow with Psivant Sciences.

## Supplementary Material

DD-005-D5DD00464K-s001

## Data Availability

All of the code required to reproduce the results reported in this study can be found on GitHub: https://github.com/MobleyLab/active-learning-notebooks/blob/main/MultiobjectiveAL.ipynb as well as permanently archived on Zenodo: https://doi.org/10.5281/zenodo.17180098. Docking scores from the DOCKSTRING benchmark are available at https://figshare.com/articles/dataset/dockstring_dataset/16511577. Supplementary information (SI) is available. See DOI: https://doi.org/10.1039/d5dd00464k.
