# Supplementary material for: A simple compound prioritization method for drug discovery considering multi-target binding
Source: Digit Discov. 2026 Feb 10;5(3):1204–14. doi: 10.1039/d5dd00464k (PMC12888084; doi:10.1039/d5dd00464k)
Supplement: DD-005-D5DD00464K-s001 [file DD-005-D5DD00464K-s001.pdf]

Supplementary Information (SI) for Digital Discovery.
This journal is © The Royal Society of Chemistry 2026

Supporting Information for: "A simple compound prioritization method for drug discovery considering multi-target binding"

Alžbeta Kubincová and David L. Mobley

January 7, 2026

1 Comparison of molecular descriptors

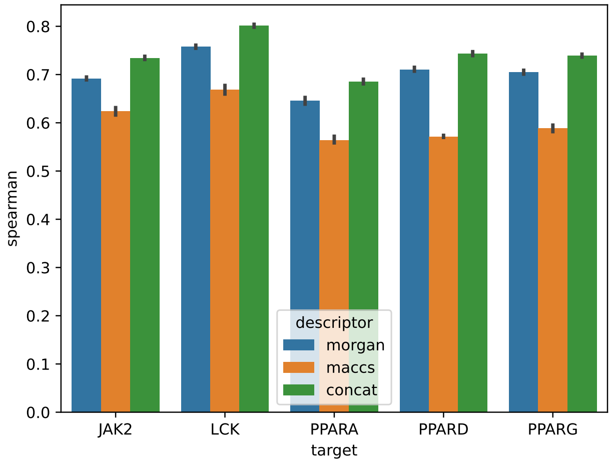

Figure 1: Test set correlation (Spearman's ρ) from GP models trained on 1000 randomly selected molecules from DOCKSTRING, using different sets of descriptors. Error bars are reported as confidence intervals over 5 repeats, initialized from different random train-test splits. The models were trained on the docking scores of each of the five targets. The Morgan fingerprints which were used throughout the Main article (chiral, 1024 bits, radius = 4) are compared with MACCS keys (computed using the `MACCSkeys` module from RDkit), and a concatenation of the Morgan fingerprint with MACCS keys. The concatenated representation leads to the most predictive models for all five targets.

2 Reducing the initial batch size

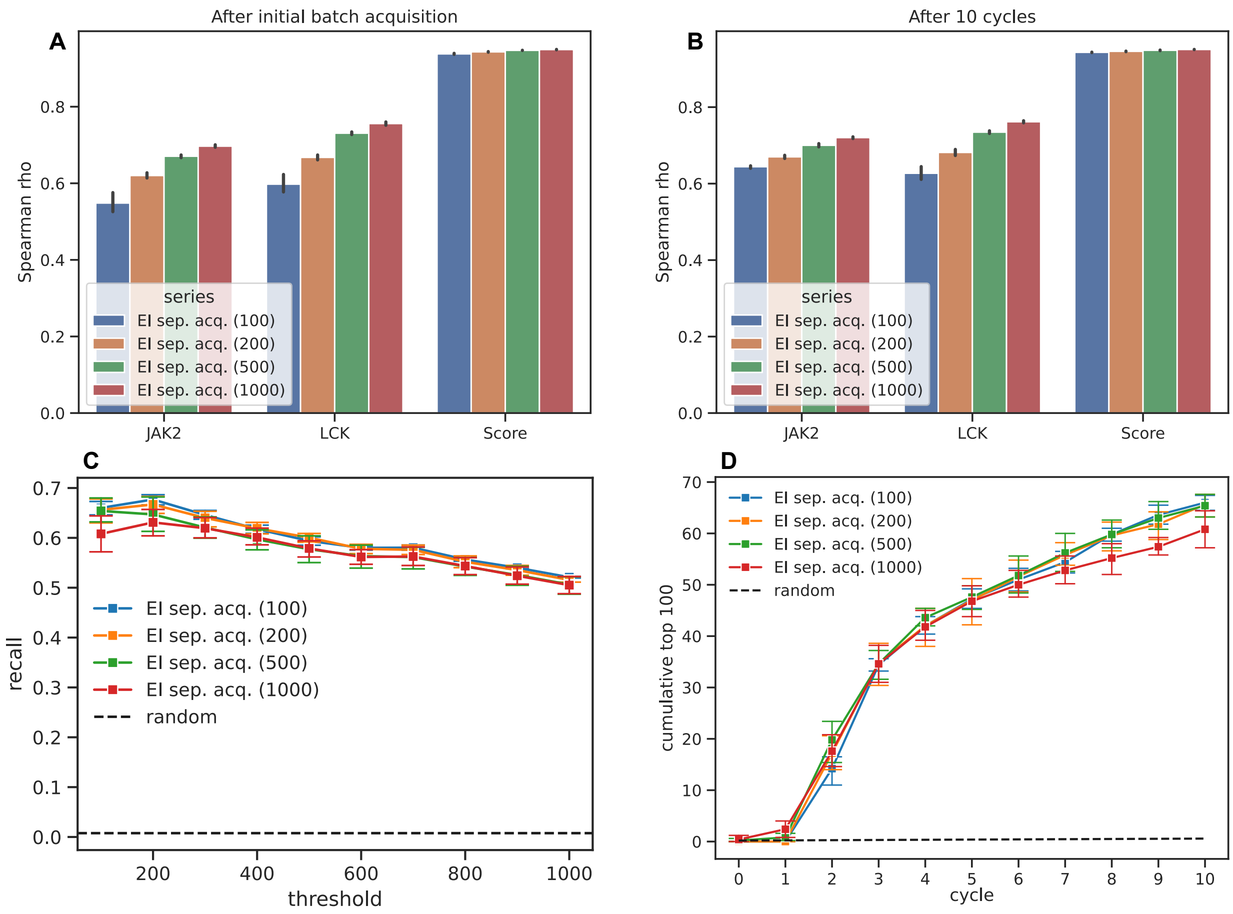

Figure 2: Initialization of the workflow with fewer ligands (100, 200, 500) compared to the 1000 initial samples used in the Main text, and the influence of the initial batch size on model correlation (A-B) and recall (C-D). A: Spearman correlation measured on predictions of compounds which stayed in the pool after 10 cycles, where the docking scores were predicted with the initial model trained only on 100-1000 samples. B: Same as A, except that the predictions were made with the final model after 10 cycles (trained on 3000 additional samples distributed over JAK2 and LCK). The improvement of JAK2 predictions after 10 cycles can be attributed to the more frequent acquisition of JAK2 scores compared to LCK (Fig. 5A in the Main article). C: Fraction of active compounds retrieved for the JAK2 system, as a function of the threshold rank for classifying a compound as active. The recall is calculated as the number of actives found within the 10 cycles, divided by the corresponding threshold. D: Retrieval of the top 100 compounds for the JAK2 system as a function of the AL cycle. This data shows that a predictive model for JAK2 and LCK can be trained on as little as 100 ligands from the DOCKSTRING dataset, and using this model in AL leads to retrieving a similar fraction of active compounds from the pool compared to the model trained on 1000 samples.

3 Reducing the number of compounds acquired per cycle

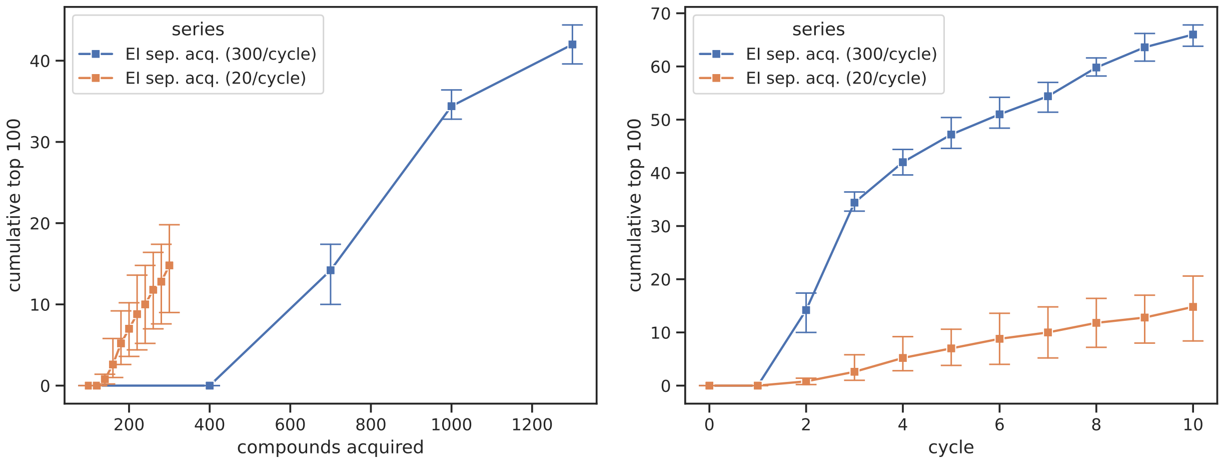

Figure 3: Comparison of batch sizes for the JAK2/LCK system. The plots show the retrieval of the top 100 compounds as a function of the total number of compounds acquired (left) or the number of AL cycles performed (right). Here, an initial batch of 100 ligands was chosen at random to warm up the ML model (instead of 1000 ligands in the Main article). We compare acquiring 300 scores in each cycle, which was the option used in the Main article, with the acquisition of only 20 scores per cycle. The smaller batch size results in a higher fraction of top compounds in the selected ligands (left), but at the same time requires more cycles, limiting the potential to parallelize this approach.
